# Towards a knowledge graph for pre-/probiotics and microbiota–gut–brain axis diseases

**DOI:** 10.1038/s41598-022-21735-x

**Published:** 2022-11-08

**Authors:** Ting Liu, Gongjin Lan, K. Anton Feenstra, Zhisheng Huang, Jaap Heringa

**Affiliations:** 1 Department of Computer Science, Center for Integrative Bioinformatics, Vrije Universiteit Amsterdam, 1081 HV Amsterdam, The Netherlands; 2 Knowledge Representation and Reasoning Group, Department of Computer Science, Vrije Universiteit Amsterdam, 1081 HV Amsterdam, The Netherlands; 3 Department of Computer Science and Engineering, Southern University of Science and Technology, Shenzhen, 518055 China

**Keywords:** Biotechnology, Computational biology and bioinformatics, Microbiology

## Abstract

Scientific publications present biological relationships but are structured for human reading, making it difficult to use this resource for semantic integration and querying. Existing databases, on the other hand, are well structured for automated analysis, but do not contain comprehensive biological knowledge. We devised an approach for constructing comprehensive knowledge graphs from these two types of resources and applied it to investigate relationships between pre-/probiotics and microbiota–gut–brain axis diseases. To this end, we created (i) a knowledge base, dubbed ppstatement, containing manually curated detailed annotations, and (ii) a knowledge base, called ppconcept, containing automatically annotated concepts. The resulting Pre-/Probiotics Knowledge Graph (PPKG) combines these two knowledge bases with three other public databases (i.e. MeSH, UMLS and SNOMED CT). To validate the performance of PPKG and to demonstrate the added value of integrating two knowledge bases, we created four biological query cases. The query cases demonstrate that we can retrieve co-occurring concepts of interest, and also that combining the two knowledge bases leads to more comprehensive query results than utilizing them separately. The PPKG enables users to pose research queries such as “*which pre-/probiotics combinations may benefit depression?*”, potentially leading to novel biological insights.

## Introduction

Knowledge graphs have been widely used in biomedical domain applications such as comorbidity analysis^1^, disease classification^2^, drug discovery^3^, drug prioritization^4^ and target prediction^5^. They enable researchers to identify new promising effects of drugs^5^, discover potential drug action mechanisms^6^, predict unknown adverse drug reactions^7^, or project potential side-effects of drug–drug interactions^8,9^. Knowledge graph technology is useful for integrating and querying heterogeneous data across multiple databases and biomedical literature. In our previous studies^10,11^, we constructed a knowledge graph, named MiKG, to discover potential relations between gut microbiota, neurotransmitters and mental disorders by manually extracting and normalizing relevant entities and relations in publications. For the current study, we constructed a vastly larger knowledge graph to integrate the knowledge from the biomedical literature by using a two-level approach that combines much more in-depth manual relational curation by a panel of experts with automatic concept annotation.

Gut microbiota are known to have a profound impact on the communication between the central nervous system and the enteric (gut) nervous system^12,13^. These interactions are referred to as the microbiota–gut–brain (MGB) axis^14^. Dysfunction of the MGB axis is associated with a number of human diseases, including obesity, diabetes and mental disorders^15,16^. Improving the gut microbiome could therefore be a useful approach for treating diseases in which the MGB axis is involved^17^. As modulators of gut microbiota, prebiotics and probiotics are widely used to alter the gut microbial communities for beneficial effects^18^. The biomedical literature contains abundant knowledge regarding the beneficial effects of specific prebiotics and probiotics on MGB-axis diseases. However, papers are usually written by humans for humans and are thus not structured in a machine-accessible format that may support semantic integration and querying. In this work, we aim to integrate and normalize the unstructured literature data into a pre-/probiotics knowledge graph (PPKG) through the two-level approach: manually extract detailed annotations and automatically identify broadly covered concepts, which are incorporated in two separate knowledge bases: ppstatement and ppconcept, respectively, for easy access and management.

To develop the ppstatement knowledge base, we constructed detailed annotations from manually extracted entities, attributes and relations from 134 selected articles. The ppconcept knowledge base, tuned for broad coverage of concepts, was obtained by using an automated concept identifier^19^ that was able to rapidly recognize community-accepted concepts from 29,492 retrieved articles. The detailed annotations and the relationship between recognized concepts and the source articles are represented in triplet format: subject–predicate–object^20^. Furthermore, the extracted entities and recognized concepts are linked to the existing entity descriptions in the UMLS, MeSH and SNOMED CT databases with Uniform Resource Locators (URLs). These three databases hold community-accepted and relevant medical terms. We imported the two knowledge bases together with the aforementioned three databases into GraphDB to complete the data integration of the PPKG and carry out semantic searches. The thus constructed PPKG presents the effects of prebiotics and probiotics on human diseases in both machine-accessible and human-readable formats.

We present four biological query cases to validate the performance of PPKG and demonstrate the added value of integrating the two knowledge bases. The queried results indicate that PPKG enables users to retrieve direct relations and infer novel relations. The established resources and findings can provide an efficient way to retrieve associations of diseases of interest, and offer novel and meaningful biological insights. The main contributions of this research can be summarized as follows:

1. a two-level approach that combines manual relational curation with automated concept annotation;
2. a method to investigate relationships between pre-/probiotics and microbiota-gut-brain axis diseases;
3. a knowledge graph containing well-structured knowledge about prebiotics and probiotics.

## Methods

**Figure 1:**
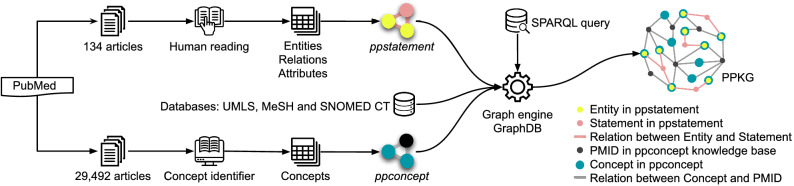
The workflow of the two-level construction process for PPKG. First, detailed annotations are manually extracted from 134 selected articles to construct the ppstatement knowledge base. Second, broadly covered concepts are automatically annotated from 29,492 retrieved articles to form the ppconcept knowledge base. The two knowledge bases are integrated with the other databases as PPKG in GraphDB, which supports accessing and reasoning over data with SPARQL query language.

We constructed the PPKG by combining manual relational curation and automatic concept annotation. Specifically, we manually extracted detailed annotations from 134 articles for the ppstatement knowledge base (see “Manual curation of the ppstatement knowledge base” section) and automatically annotated broadly covered concepts by using a concept identifier^19^ for the ppconcept knowledge base (see “Automatic generation of the ppconcept knowledge base” section). Both knowledge bases are stored in the Resource Description Framework (RDF) triple format. We imported them into an RDF triple store (i.e. GraphDB database) along with the MeSH (version 2020), UMLS (version 2020AA Full Release) and SNOMED CT (version 20180802) databases. We chose the open-source GraphDB database platform as our current store because of its high performance and industry acceptance. GraphDB provides the core infrastructure for data integration, relational exploration, and graph visualization. In addition, it allows users to calculate the data expansion ratio, which is the ratio of the inferred triples to the explicit triples under RDFS reasoning. The data in GraphDB can be accessed and reasoned over by using the SPARQL query language. Furthermore, the visualization of data structures and query results for the current study was implemented through Cytoscape^21^ (version 3.9.1; https://cytoscape.org/). The workflow of the two-level construction process for PPKG is shown in Fig. 1.

**Table 1:** Hierarchical overview of annotations in the ppstatement knowledge base.

Class	Element	Description
Entities	Species	Model organism (e.g. human, mouse, rat or fruit fly)
	Population	Research object with or without diseases (e.g. pregnancy, obesity, depression or stress)
	Sex	Gender of the model organism (e.g. male, female or random mixing)
	Probiotics	Bacterial strains that used for treatment (e.g. *L. brevis* or *B. bifidum*)
	Prebiotics	Prebiotics compounds that used for treatment (e.g. FOS or GOS)
	Body part	The body part where the treatment effect occurs (e.g. cortical, plasma or hippocampus)
	Reference	The articles that the facts were extracted, presented by PMIDs and DOIs
Attributes	Treatment time	Duration of the treatment
	Regulation	Up-regulate, down-regulate or no effect
	Treatment effect	The entity is affected by the treatment (e.g. depression score or gene expression)
Relations	Owl:sameAs	Used to align co-entities between different data sets
	rdf:hasProperty	Used to define additional properties with a domain
	rdfs:subClassOf	Used to state that one class is a subclass of another

### Manual curation of the ppstatement knowledge base

To construct the ppstatement knowledge base, we retrieved relevant literature from PubMed using the keyword combinations of <probiotics, prebiotics, gut microbiota> and <microbiota–gut–brain axis, MGB axis, human disease>. We selected 134 articles that described MGB-axis disease treatment with probiotics and/or prebiotics considering the relevance, such as citation, publication date, and keywords in the titles and abstracts of the articles as well. We reviewed the full text of all 134 articles and manually listed the entities, attributes and relations of interest. For example, consider the sentence “Oral administration of *Lactobacillus (L.) rhamnosus* for 8 weeks reduced depression scores in patients with depressive disorder”, from which we can extract the entities including species: human, population: depressive disorder; probiotics: *L. rhamnosus*; and attributes such as treatment time: 8 weeks; regulation: down; treatment effect: depression score. The extracted data is structured in the ppstatement format that consists of entities, attributes and relations. The relations between statements and entities, as well as statements and attributes, are described using rdf:hasProperty term. For all the extracted data from the 134 articles, the entities may consist of species, population, sex, probiotics, prebiotics, body part and reference, as described in Table 1. These entities make up the terminology components (TBox), while statements act as assertion components (ABox) which make use of the entities. The elements of attributes involve treatment time, regulation and treatment effect. Relations contain three elements of owl:sameAs, rdf:hasProperty and rdfs:subClassOf. Additionally, entities are linked to the UMLS, MeSH and SNOMED CT databases using URLs.

**Figure 2:**
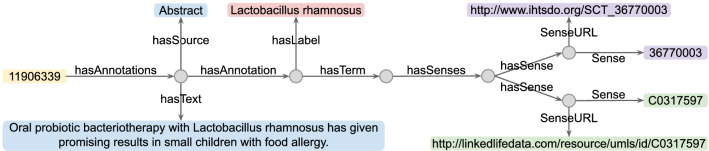
Illustration of the structure of ppconcept knowledge base. The yellow rectangle indicates the PMID. Gray dots are customized strings. Blue rectangles are free texts and the red one is the label of the annotated concept. Purple and green rectangles represent URLs from the SNOMED CT and UMLS databases, respectively.

### Automatic generation of the ppconcept knowledge base

We retrieved a total of 29,492 articles from PubMed by using the keywords probiotics and/or prebiotics. We used the concept identifier by Aït-Mokhtar et al.^19^ to annotate the concepts in the titles and abstracts of the retrieved articles (see the workflow in Fig. 1). It recognizes concepts in free text using the UMLS and SNOMED CT vocabularies as the default term databases. We described the relations between recognized concepts and source articles in triplet format. Figure 2 depicts a representative example of the structure of the ppconcept knowledge base, in which the ‘*L. rhamnosus*’ annotated as a concept from the paper-text “Oral probiotic bacteriotherapy with *L. rhamnosus* has given promising results in small children with food allergy.” Additionally, the concept ‘*L. rhamnosus*’ has sense URLs from the UMLS and SNOMED CT databases. Furthermore, the terms ‘Oral’, ‘Small’, ‘Children’, ‘Food’, ‘Allergy’ and ‘Food allergy’ are all recognized as concepts in this paper-text. Overall, an article contains a number of paper-texts, each of which is constructed with multiple concept annotations, and each concept is linked to the UMLS and SNOMED CT databases using URLs.

### Knowledge base integration and data access

We integrated the ppstatement, ppconcept, MeSH, UMLS and SNOMED CT databases into GraphDB, which connects independent knowledge bases and indexes datasets for semantic search, to complete the PPKG construction. The data in GraphDB can be accessed and reasoned over by using the SPARQL query language^22^, which is a popular query protocol for retrieving and manipulating structured data in RDF format^23^. We used the SELECT query form to set up semantic queries. This query form returns result sets that can be exported as *json*, *xml*, *csv* and *pdf* files. In writing SPARQL queries, namespace declarations allow using a prefixed name instead of an Internationalized Resource Identifier. Therefore, we have defined a set of namespace prefix bindings to start queries. For example, we used the prefixed name ppconcept instead of <http://wasp.cs.vu.nl/ppconcept#> and ppstatement instead of <http://wasp.cs.vu.nl/ppstatement#>. The variables to be returned were defined using the select distinct statement, and the query conditions were specified using the where clause. We used the filter condition to limit data output based on some criteria. Furthermore, the union, intersect and except clauses were used to combine or exclude similar rows from multiple tables. For example, we used the union clause to combine the results of two separate queries from ppstatement and ppconcept into a single set. We have provided several detailed query protocols in the Supplementary Information document.

## Results

### Concepts and triplet statistics in the knowledge graph

**Figure 3:**
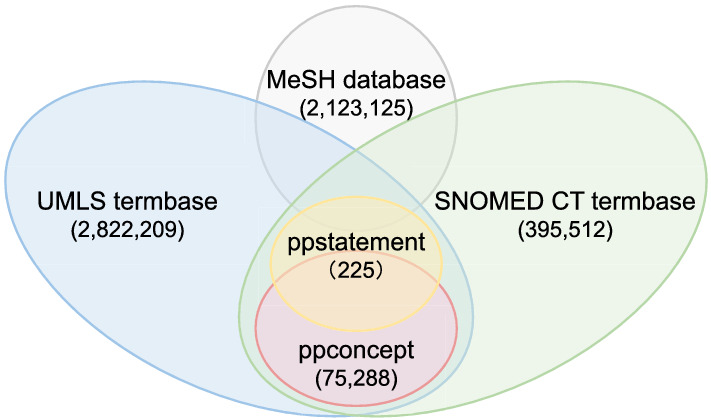
Relation of entities and concepts between the databases and the knowledge bases. Most of the entities in ppstatement are covered by ppconcept, which are matched to the UMLS and SNOMED CT databases with URLs. Additionally, entities in ppstatement are mapped to the MeSH database as well to gain more entity descriptions.

The constructed PPKG contains two knowledge bases: a manually curated ppstatement knowledge base and an automatically generated ppconcept knowledge base. The details of their construction are described in the “Methods” section. From 134 articles, the ppstatement knowledge base comprises a total of 225 manually extracted entities, as well as their attributes and relations. The ppconcept knowledge base includes 75,288 concepts culled from the titles and abstracts of 29,492 papers selected by keywords. Most of the entities in ppstatement are covered by ppconcept (see Fig. 3), which extends them by providing broadly covered relevant concepts, while ppstatement offers more detailed annotations. The entities and concepts in both knowledge bases are matched to existing entity descriptions in the UMLS and SNOMED CT databases. The entities in ppstatement are mapped to the MeSH database by linking URLs.

For both knowledge bases, the expansion ratio is calculated as the ratio of inferred triples to explicit triples under RDFS reasoning, as shown in Table 2. In total, the ppstatement contains 5683 triples, while the ppconcept is much larger with 40,442,404 triples. Aside from the explicit relations extracted from the literature, the two knowledge bases imply inferable indirect relations. The ppstatement contains 209 inferred triples, resulting in an expansion ratio of 3.7%. The ppconcept contains 134 inferred triples, which yields a much lower expansion ratio of 0.00033%. After combining these two knowledge bases with the UMLS, MeSH and SNOMED CT databases, a total of 10,136,056 new triples could be inferred from the existing triples, resulting in a final expansion ratio of 16.5%. Integrating the two knowledge bases with other existing databases clearly brings a higher potential for discovering unseen relations.

**Table 2:** Number of triples in knowledge bases.

Knowledge base	Explicit	Inferred	Expansion ratio (%)	Total
ppstatement	5684	209	3.7	5893
ppconcept	40,442,404	134	0.00033	40,442,538
ppstatement + ppconcept	40,448,088	235	0.00058	40,448,323
ppstatement + ppconcept + UMLS + MeSH + SNOMED CT	61,577,896	10,136,057	16.5	71,713,953

The expansion ratio is defined by the ratio of inferred triples to explicit triples.

**Figure 4:**
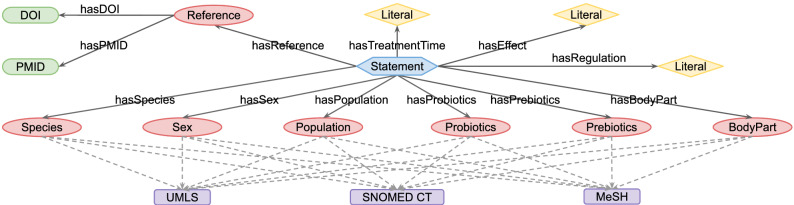
Entities, attributes and relations in ppstatement knowledge base. The blue hexagon represents assertion and the red ellipses represent terminologies. DOI and PMID (shown as green rectangles) are used as reference identifiers. Yellow diamonds are not entities but free texts. The entities are mapped to UMLS, MeSH and SNOMED CT (shown as purple rectangles) with URLs. Arrows indicate the direction of relations from source to target.

### Visualization of ppstatement and ppconcept

**Figure 5:**
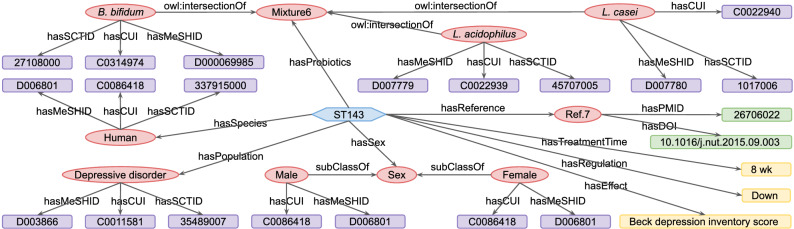
Schematic diagram of statement ST143 extracted from the ppstatement knowledge base. The blue hexagon represents assertion and the red ellipses represent terminologies. Assertion includes the ‘Statement’ (ST143). Terminologies consist of the entities: ‘Species’ (human), ‘Sex’ (male and female), ‘Population’ (depressive disorder), ‘Reference’ (Ref.^7^) and ‘Probiotics’ (*B. bifidum*, *L. acidophilus* and *L. casei*). Yellow rectangles are free texts. DOI and PMID (shown as green rectangles) are used as reference identifiers. Purple rectangles represent the IDs of entities in the UMLS, MeSH and SNOMED CT databases. Arrows indicate the direction of relations from source to target.

The visualization of a knowledge graph provides insight into its internal hierarchies and connections. In Fig. 2, we illustrate the internal structure of the ppconcept knowledge base with a representative example. In general, a paper-text consists of multiple concept annotations, and each concept is mapped to the UMLS and SNOMED CT databases. Furthermore, we depict the structure of the ppstatement knowledge base in Fig. 4, in which the ppstatement is represented as a collection of TBox and ABox, as explained in “Manual curation of the ppstatement knowledge base” section. ABox includes the assertion ‘Statement’, and TBox consists of the terminologies: ‘Species’, ‘Sex’, ‘Population’, ‘Prebiotic’, ‘Probiotic’, ‘BodyPart’ and ‘Reference’. Multiple triples are involved in one statement. There are 446 statements in the ppstatement knowledge base. We here select a representative statement ST143 as an example for a detailed explanation, as shown in Fig. 5, from which the contents of ST143 can be summarized as follow: An 8-week combination treatment of probiotic strains *Bifidobacterium (B.) bifidum*, *L. acidophilus* and *L. casei* reduced the Beck Depression Inventory scores of depressed patients. This statement is derived from the article with a PMID of 26706022 and a DOI of 10.1016/j.nut.2015.09.003. Furthermore, concepts have their URLs in the MeSH, UMLS and SNOMED CT databases. For example, the probiotic strain *B. bifidum* has CUI: C0314974, MeSH ID: D000069985 and SCT ID: 27108000. Integrating the two knowledge bases with these existing databases allows us to align entities and obtain extended knowledge about the terms.

**Figure 6:**
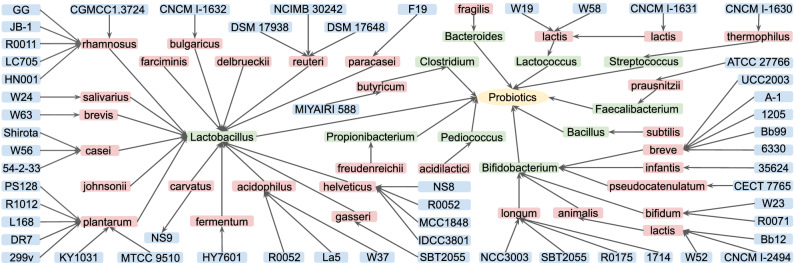
Hierarchy of probiotic strains in ppstatement knowledge base. Arrow lines indicate relations of ‘rdfs:subClassOf’. Green, red and blue rectangles represent the probiotic genera, species and strains, respectively.

We captured the hierarchical taxonomic relations of probiotic strains in the PPKG knowledge base, as shown in Fig. 6. In general, scientists look into the benefits of probiotics at the strain level because different strains carry different benefits. For example, *L. reuteri* DSMZ 17648 has been used to control *Helicobacter pylori* in humans^24^, whereas *L. reuteri* NCIMB 30242 supports heart health by naturally maintaining healthy cholesterol levels^25^. Figure 6 shows that the majority of probiotic strains belong to the bacterial genera *Lactobacillus* and *Bifidobacterium*, with the rest coming from the genera *Propionibacterium*, *Clostridium*, *Bacteroides*, *Lactococcus*, *Streptococcus*, *Bacillus*, *Faecalibacterium* and *Pediococcus*. Detailing probiotics at the strain level allows for strain-specific queries from the PPKG.

**Figure 7:**
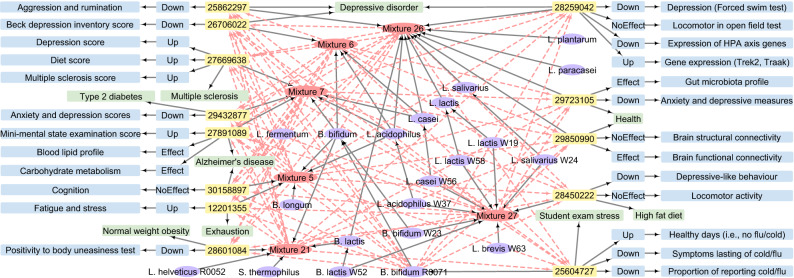
The returned results of *B. bifidum* strains’ effects on diseases by query case 1, with the code shown in SI Listing 1. Yellow rectangles: PMIDs; green rectangles: populations; red ellipses: probiotic mixtures; purple ellipses: probiotic strains; blue rectangles: treatment effects; solid black lines: direct relations; red dotted lines: indirect relations between articles.

### Validating the practicality and effectiveness of PPKG

We show the validity and usefulness of the PPKG with two use cases: one to gain knowledge on the health effects of specific probiotic strains, and the other to obtain statements about the benefits of probiotics in treating specific diseases.

Query case 1 queries the ppstatement knowledge base for all diseases influenced by *B. bifidum*. SI Listing 1contains the query code for case 1, from which we obtained 13 related articles, as shown in Fig. 7. The query result contains a total of 114 direct relations and 78 indirect relations between articles, yielding an expansion ratio of 68%. We can conclude from the direct relations that *B. bifidum*, when combined with other probiotic strains, has effects on a wide range of conditions, including depressive disorder, type-2 diabetes, multiple sclerosis, Alzheimer’s disease, obesity, exhaustion, and a high-fat diet. Furthermore, these mixtures influence blood lipids and gut microbiota profiles, as well as brain connectivity and gene expression of some specific genes. Aside from these combinations, *B. bifidum* R0071 can be used alone for the treatment of colds and flu. In addition to the direct relations, the indirect relations (red dotted lines) suggest alternative beneficial combinations of *B. bifidum* and other probiotic strains, and may provide benefits when used alone.

**Figure 8:**
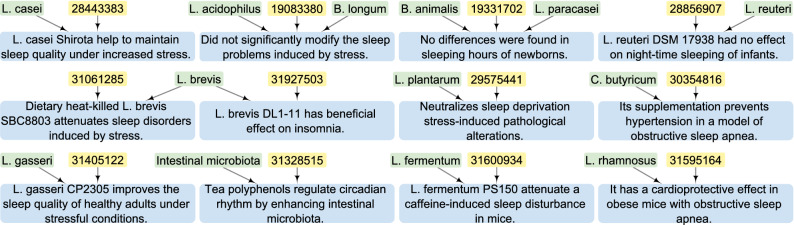
The retrieved paper-texts that contain children concepts of *Microorganisms* and *Finding related to sleep* by query case 2, with the code shown in SI Listing 2. Yellow rectangles: PMIDs; green rectangles: annotated concepts; blue rectangles: free texts. For clarity of presentation, long sentences were simplified and probiotic strains were abbreviated.

Query case 2 queries the ppconcept knowledge base for all probiotics that impact sleep conditions. Using the query code for case 2 (see SI Listing 2), we retrieved 12 relevant paper-texts co-occurring with the children of SNOMED CT concepts *Microorganisms* (SCTID:264395009) and *Finding related to sleep* (SCTID:106168000). According to these paper-texts, a variety of probiotic strains have positive effects on sleep conditions, as can be seen from Fig. 8. For example, *L. casei*, *L. brevis* and *L. gasseri* improve sleep quality during a period of increasing stress. Aside from that, *L. plantarum*, *Clostridium (C.) butyricum* and *L. rhamnosus* help to alleviate the pathological alterations caused by various sleep conditions. Furthermore, certain strains have been shown to be non-beneficial for sleep disorders. For example, the combination of *B. longum* and *L. acidophilus* has no discernible effect on the sleep problems caused by stress. These paper-texts imply that we can obtain direct relations from the ppconcept knowledge base.

Overall, these two query cases evidence both the health effects of *B. bifidum* in treating various diseases and the benefits of multiple probiotics in treating sleep conditions, demonstrating the practicality and effectiveness of PPKG.

**Figure 9:**
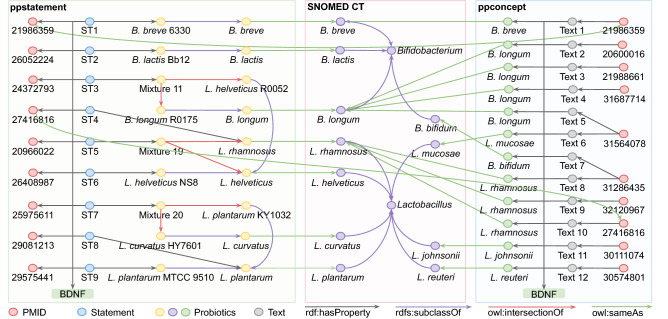
The returned relations between probiotics and the BDNF by query case 3, and the code is provided in SI Listing 3. Arrows indicate the direction of relations from source to target.

**Figure 10:**
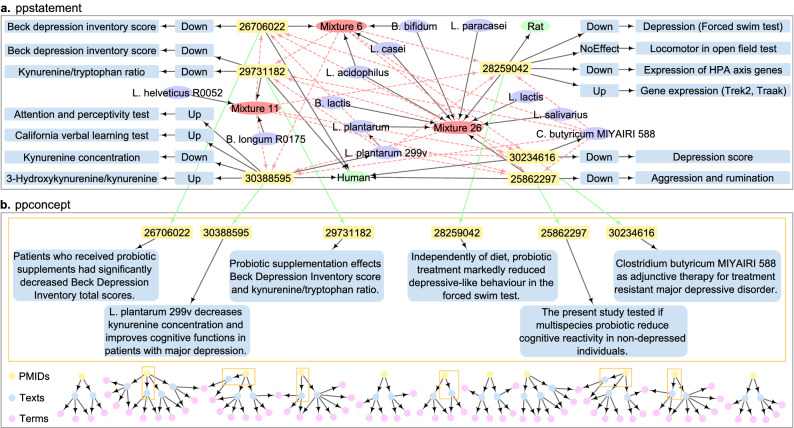
The returned results of probiotics’ effects on depressive disorder by query case 4, of which the code is reproduced in SI Listing 4: (**A**) Assertions from six papers in ppstatement; (**B**) PMIDs in ppconcept overlapping with ppstatement, and associated paper-texts. The bottom part indicates 333 further relevant texts in ppconcept, which do not overlap with ppstatement. Green ellipses: model organisms; purple ellipses: probiotic strains; red ellipses: probiotic mixtures; yellow rectangles: PMIDs; green lines: same PMIDs; black lines: direct relations; red lines: indirect relations between articles.

### Added value of integrating ppstatement and ppconcept

In this section, we present two further query cases to demonstrate the added value of integrating the ppstatement and ppconcept bases with other existing databases.

First, we create query case 3 to retrieve probiotics that influence the expression of the Brain-Derived Neurotrophic Factor (BDNF) gene. The query codes are shown in SI Listing 3, which returns 17 unique articles, nine from ppstatement and ten from ppconcept, with two overlapping, as shown in Fig. 9. The nine articles in ppstatement show that BDNF levels increase upon a single or combined treatment with several specific probiotic strains. We retrieved 12 paper-texts from ppconcept in which the concepts of probiotics and BDNF co-occurred. Furthermore, Fig. 9 shows that the entities and concepts in both knowledge bases are mapped to the SNOMED CT database.

We design query case 4 to gain knowledge on a specific disease that may be influenced by probiotics, i.e. query all probiotics that impact depression. Using the query code shown in SI Listing 4, we retrieved 208 articles, six from the ppstatement and 202 from the ppconcept, where the articles from ppstatement are also covered by ppconcept (see Fig. 10). From the six articles in ppstatement, we obtained 13 assertions expressing that probiotics have effects on depression Fig. 10a, and 339 paper-texts where the children of concepts *Microorganisms* and *Depressive disorder* co-occur from the ppconcept knowledge base (see the bottom part of Fig. 10b). An example of where ppstatement yields a clear benefit over ppconcept can be seen in the results for PMID 25862297 (in the bottom right of both panels). In ppstatement, manual curation allows complex information from multiple sentences across the paper to be integrated, for example, explicit sets of probiotic mixtures could be linked to the reduction of aggression and rumination, which is associated with depression. In contrast, in ppconcept atomic information was extracted, Fig. 10b shows one such phrase for each PMID as an example. The other five overlapping PMIDs indicate that the automatic concept annotation in ppconcept is also able to identify some of the relevant relations. Thus, the query case shows that the information retrieved from the unique elements contained in each of the two knowledge bases can be leveraged by mapping the overlapping PMIDs. Although outside of the scope of the current example, the 333 remaining paper-texts may be of interest for further scrutiny. For example, we obtained paper-texts such as “Combination of *L. helveticus* R0052 and *B. longum* R0175 reduces post-myocardial infarction depression symptoms and restores intestinal permeability in a rat model” from PMID 21933458 and “*L. mucosae* and *B. longum* synergistically alleviate immobilization stress-induced anxiety/depression in mice by suppressing gut dysbiosis” from PMID 31564078, which represent biological research facts and are open to further review. Thus, we demonstrate that integrating the two knowledge bases yields more comprehensive query results than utilizing them independently.

The query results from ppstatement generate 52 direct relations and 24 indirect relations between articles, giving an expansion ratio of 44%. From these relations, we see that several probiotic strains have been studied either as a single-strain or in mixtures for the treatment of humans and rats, and have been shown to reduce depression scores, gene expression, kynurenine biochemistry, and attention in depressed patients. In addition to the direct relations, we can draw a few conclusions from the indirect relations: a new combination of *C. butyricum* MIYAIRI 588, mixture 6, and mixture 11 may, as they all contribute to lower depression scores, may improve the condition of depressed patients more than when using them alone. Such inferred relations appear biologically plausible and could be of interest to the biological community.

### User-defined repository and queries

The knowledge bases used in this study are available on the project website (https://wasp.cs.vu.nl/MicroUniverse/download/ppkg). We also provide a README file that explains how to integrate multiple knowledge bases into the PPKG in GraphBD step-by-step. In short, in GraphDB, the first step is to create a new repository called PPKG. Then, download the ppstatement.ttl and ppconcept.zip databases from the website and import them into the repository by setting them as named graph with URLs: http://wasp.cs.vu.nl/ppstatement and http://wasp.cs.vu.nl/ppconcept, respectively. Other custom knowledge bases and existing online databases can also be loaded into the repository in the same way. Data in GraphDB can be accessed and reasoned about using the SPARQL protocol.

We provide several query protocols as used in the query cases discussed above. They are written in the SPARQL query language and are presented in the Supplementary Information file, which can also be accessed through the project website. Users can easily create their queries by using the provided query protocols as templates and changing parameters or extending the query as needed. Each template is followed by a detailed explanation of where, when, and how to change the parameters. The query protocols used here are all composed of basic query clauses, and each protocol contains less than 30 variables, resulting in a time of less than half a minute to retrieve data from the knowledge graph. Certainly, keeping queries simple and efficient, filtering down select statements, and limiting output results can improve the efficiency of queries.

## Discussion

We transformed the data in scientific publications into both computer-processable and human-readable formats to construct the PPKG for investigating the effects of pre-/probiotics on MGB-axis diseases, as a proof of concept for our two-level approach to combining human- and machine-based information retrieval.

We created the ppstatement knowledge base with rich detailed annotations and the ppconcept knowledge base with numerous automatically derived concepts. As shown in Figs. 7 and 10, the ppstatement knowledge base returns detailed results for our semantic queries. However, since the knowledge in the ppstatement is more detailed but incomplete, we remedy this by creating the ppconcept knowledge base using automatic concept annotation with the aim of including a large body of literature and concepts. As shown by Figs. 8 and 10, the ppconcept knowledge base enables us to obtain relevant articles (related paper-texts and concepts) from a vast literature via semantic queries, as can be seen from Figs. 8 and 10. As a result, these two knowledge bases are complementary, and they work together to ensure the accuracy and completeness of the query results.

We visualized the internal structure of the integrated PPKG database by first illustrating the connections within the ppconcept knowledge base with a representative example in Fig. 2, which shows the way we link annotated concepts and source articles. We then provided the hierarchies within the ppstatement knowledge base, as shown in Fig. 4, which displays the relationships between assertions and terminologies, as well as attributes associated with them. We further choose a representative statement (i.e. ST143) to detail the schematic diagram of statements, as shown in Fig. 5. We additionally showed the relationships of probiotic strains from the ppstatement knowledge base in Fig. 6.

We validated the practicality and effectiveness of our PPKG as a means of integrating knowledge from multiple data sources and different levels of description, as described in “Validating the practicality and effectiveness of PPKG” section. Using the PPKG, we can retrieve detailed annotations that contain direct and indirect relations, as well as paper-texts that cover the concepts of interest from a large body of literature (see Figs. 7, 8). We also illustrated the added value of integrating the knowledge bases at different levels of description, as shown in “Added value of integrating ppstatement and ppconcept” section. Aligning entities and concepts across databases produces rich query results and indirect relations, as can be seen in Fig. 9. As a more specific use case, Fig. 10 shows that while ppstatement provides more detailed statements than ppconcept, the latter leads to a larger collection of concepts and paper-texts. Furthermore, compared to our previous MiKG^10,11^, which was constructed using only manual curation, PPKG provides both richer manually extracted relations and many more automatically annotated concepts. We thus conclude that integrating the two knowledge bases leads to richer query results than what could be obtained when using them separately.

The PPKG generally allows users to retrieve multiple types of queries: (1) the core entities and class relationships of prebiotics and probiotics; (2) the relationship between probiotics and diseases, as demonstrated by the four query cases presented in “Validating the practicality and effectiveness of PPKG” and “Added value of integrating ppstatement and ppconcept” sections; (3) the equivalent link information between databases, as demonstrated by the linkage of *B. longum* across datasets and its related information in Figs. 7 and 9; (4) paper-texts in which interested concepts co-occur. Users can relate a concept from a specific work with other concepts from the same study, as well as with concepts from similar investigations. To apply the two-level approach to other domains, several components are needed: (1) software (such as a concept identifier) to annotate concepts and build the ppconcept graph; (2) an ontology database to relate concepts in papers to existing ontologies (akin to the ppconcept graph); (3) more complex and multiple-concept statements captured in a network fashion (similar to the ppstatement graph).

Despite the fact that we can retrieve meaningful and interesting results from the PPKG, this study has some limitations in relational extraction and semantic retrieval. In this study, we construct the PPKG using a two-level approach that combines manual curation with automatic concept annotation. Manual extraction of relations ensures accuracy but is time-consuming when dealing with large amounts of literature. Furthermore, the concept identifier is capable of recognizing concepts from large amounts of literature but is unable to extract the relations between concepts and their attributes. As a next step, we will incorporate natural language processing techniques to extend the concept identifier with the extracted relational annotations as a template, thereby enabling it to auto-extract the relations from the vast literature^26^. Further possible extensions could include the algorithmic analysis of correctness and discrepancy of graphical data, e.g. using network representation learning or heuristic algorithms^27,28^. In addition, we stored the databases on a local system and accessed the data using the SPARQL query language. This approach, however, may not be end-user-friendly. End-users from the biomedical community may not be sufficiently skilled at writing SPARQL queries, not to mention understanding semantic databases. We therefore will create an endpoint with visualization so that users do not have to process the query results locally, as well as provide simple input forms based on available query templates. Both enhancements will facilitate the construction and application of biomedical knowledge graphs.

## Conclusion

Current literature on MGB-axis diseases contains a wealth of potential relations between the use of pre-/probiotics and the treatment of these diseases. To leverage this information, we need to perform queries on the relational data of entities, attributes, and concepts across various literature datasets. Data in the literature, however, is structured for human reading rather than machine access. This research reduces the gap between human-readable and machine-accessible data sources by developing a knowledge graph based on two complementary knowledge bases: a manually curated ppstatement knowledge base with rich detailed annotations, and an automatically generated ppconcept knowledge base with broadly covered concepts.

We designed four query cases to validate the performance of the developed knowledge graph. The results of executing these query cases show that our two-level PPKG can be used to retrieve potential relations between pre-/probiotics usage and the treatment of MGB-axis diseases. Users can use the PPKG to ask questions at different levels of detail, from very general (like “which probiotics benefit mental health”) to completely specific (e.g. “does *B. bifidum* have an effect on BDNF”). Finally, we presented how our knowledge graph technology can be used to discover novel relations, which can lead to new ideas for MGB-axis disease treatment with probiotics and/or prebiotics. Our approach for constructing a two-level knowledge graph may be useful for other diseases as well, while the query cases (and query codes) included in this work should be helpful for retrieving the associations for such other diseases of interest.

## Supplementary Information

Supplementary Information.

## Data Availability

Data sets are available at https://wasp.cs.vu.nl/MicroUniverse/download/ppkg/.
