# Supplementary material for: Towards a knowledge graph for pre-/probiotics and microbiota–gut–brain axis diseases
Source: Sci Rep. 2022 Nov 8;12:18977. doi: 10.1038/s41598-022-21735-x (PMC9643397; doi:10.1038/s41598-022-21735-x)
Supplement: Supplementary file 1 — Supplementary Information. [file 41598_2022_21735_MOESM1_ESM.pdf]

# Towards a knowledge graph for pre-/probiotics and microbiota-gut-brain axis diseases

**Ting Liu[1,2], Gongjin Lan[3], K. Anton Feenstra[1], Zhisheng Huang[2], and Jaap Heringa[1,*]**

[1]Center for Integrative Bioinformatics, Department of Computer Science, Vrije Universiteit Amsterdam, 1081 HV, Amsterdam, The Netherlands
[2]Knowledge Representation and Reasoning Group, Department of Computer Science, Vrije Universiteit Amsterdam, 1081 HV, Amsterdam, The Netherlands
[3]Department of Computer Science and Engineering, Southern University of Science and Technology, 518055, Shenzhen, China
[*]j.heringa@vu.nl

## SPARQL protocols for query cases

**SI Listing 1: The SPARQL protocol for query case 1: retrieve the health effects of *B. bifidum* treatment**

**SI Listing 2: The SPARQL protocol for query case 2: retrieve all probiotics that impact sleep conditions**

**SI Listing 3: The SPARQL protocol for query case 3: retrieve probiotics that affect BDNF gene expression**

**SI Listing 4: The SPARQL protocol for query case 4: retrieve all probiotics that impact depressive disorder**

**SI Listing 1.** The SPARQL protocol for query case 1: retrieve the health effects of *B. bifidum* treatment

```
prefix rdf: <http://www.w3.org/1999/02/22-rdf-syntax-ns#>
prefix owl: <http://www.w3.org/2002/07/owl#>
prefix rdfs: <http://www.w3.org/2000/01/rdf-schema#>
prefix ppconcept: <http://wasp.cs.vu.nl/ppconcept#>
prefix ppstatement: <http://wasp.cs.vu.nl/ppstatement#>

select distinct ?pmid ?probiotics ?species ?population ?regulate ?effect

where {
    {?statement  ppstatement:hasProbiotics  ppstatement:Bifidobacterium_bifidum ;
                 ppstatement:hasSpecies     ?species ;
                 ppstatement:hasPopulation  ?population ;
                 ppstatement:hasRegulation  ?regulate ;
                 ppstatement:hasEffectOn    ?effect ;
                 ppstatement:hasReference   ?reference .
    ?reference   ppstatement:hasPMID        ?pmid .}
union
    {?statement  ppstatement:hasProbiotics  ?probiotics ;
                 ppstatement:hasSpecies     ?species ;
                 ppstatement:hasPopulation  ?population ;
                 ppstatement:hasRegulation  ?regulate ;
                 ppstatement:hasEffectOn    ?effect ;
                 ppstatement:hasReference   ?reference .
    ?reference   ppstatement:hasPMID        ?pmid .
    ?probiotics  rdfs:subClassOf            ppstatement:Bifidobacterium_bifidum .}
union
    {?statement  ppstatement:hasProbiotics  ?probiotics ;
                 ppstatement:hasSpecies     ?species ;
                 ppstatement:hasPopulation  ?population ;
                 ppstatement:hasRegulation  ?regulate ;
                 ppstatement:hasEffectOn    ?effect ;
                 ppstatement:hasReference   ?reference .
    ?reference   ppstatement:hasPMID        ?pmid .
    ?probiotics  a                          owl:Class ;
                 owl:intersectionOf         ?mixture .
    ?mixture     rdf:rest*/rdf:first        ?composition .
    ?mixture     rdf:rest*/rdf:first        ppstatement:Bifidobacterium_bifidum .}
union
    {?statement  ppstatement:hasProbiotics  ?probiotics ;
                 ppstatement:hasSpecies     ?species ;
                 ppstatement:hasPopulation  ?population ;
                 ppstatement:hasRegulation  ?regulate ;
                 ppstatement:hasEffectOn    ?effect ;
                 ppstatement:hasReference   ?reference .
    ?reference   ppstatement:hasPMID        ?pmid .
    ?probiotics  a                          owl:Class ;
                 owl:intersectionOf         ?mixture .
    ?mixture     rdf:rest*/rdf:first        ?composition .
    ?composition rdfs:subClassOf            ppstatement:Bifidobacterium_bifidum .}
    }
```

**SI Listing 2.** The SPARQL protocol for query case 2: retrieve all probiotics that impact sleep conditions

```
prefix rdf: <http://www.w3.org/1999/02/22-rdf-syntax-ns#>
prefix owl: <http://www.w3.org/2002/07/owl#>
prefix rdfs: <http://www.w3.org/2000/01/rdf-schema#>
prefix snomed: <http://wasp.cs.vu.nl/sct/sct#>
prefix ppconcept: <http://wasp.cs.vu.nl/ppconcept#>
prefix ppstatement: <http://wasp.cs.vu.nl/ppstatement#>

select distinct ?pmid ?text1

where {
    ?url1       rdfs:subClassOf             <http://www.ihtsdo.org/SCT_106168000> .
    ?url1       snomed:hasEnglishLabel      ?urlLabel1 .
    ?sense1     ppconcept:SenseURL          ?url1 .
    ?senses1    ppconcept:hasSense          ?sense1 .
    ?term1      ppconcept:hasSenses         ?senses1 .
    ?annot1     ppconcept:hasLabel          ?termLabel1 .
    ?annot1     ppconcept:hasTerm           ?term1 .
    ?annos1     ppconcept:hasAnnotation     ?annot1 .
    ?annos1     ppconcept:hasSource         ?source1 .
    ?annos1     ppconcept:hasText           ?text1 .
    ?pmid       ppconcept:hasAnnotations    ?annos1 .
    ?pmid       ppconcept:hasAnnotations    ?annos2 .
    ?annos2     ppconcept:hasText           ?text2 .
    ?annos2     ppconcept:hasSource         ?source2 .
    ?annos2     ppconcept:hasAnnotation     ?annot2 .
    ?annot2     ppconcept:hasTerm           ?term2 .
    ?annot2     ppconcept:hasLabel          ?termLabel2 .
    ?term2      ppconcept:hasSenses         ?senses2 .
    ?senses2    ppconcept:hasSense          ?sense2 .
    ?sense2     ppconcept:SenseURL          ?url2 .
    ?url2       snomed:hasEnglishLabel      ?urlLabel2 .
    ?url2       rdfs:subClassOf             <http://www.ihtsdo.org/SCT_264395009> .}
order by asc (?pmid)
```

**SI Listing 3.** The SPARQL protocol for query case 3: retrieve probiotics that affect BDNF gene expression

```
prefix rdf: <http://www.w3.org/1999/02/22-rdf-syntax-ns#>
prefix owl: <http://www.w3.org/2002/07/owl#>
prefix rdfs: <http://www.w3.org/2000/01/rdf-schema#>
prefix snomed: <http://wasp.cs.vu.nl/sct/sct#>
prefix ppconcept: <http://wasp.cs.vu.nl/ppconcept#>
prefix ppstatement: <http://wasp.cs.vu.nl/ppstatement#>

select distinct ?pmid ?probiotics ?composition ?url ?text1 ?urlLabel1

where {
    {?statement     ppstatement:hasEffectOn     ?effect .
    ?statement      ppstatement:hasProbiotics   ?probiotics .
    ?statement      ppstatement:hasSpecies      ?species .
    ?statement      ppstatement:hasReference    ?reference .
    ?reference      ppstatement:hasPMID         ?pmid .
    ?probiotics     owl:sameAs                  ?url .
    filter regex    (?effect,"bdnf","i")}
Union
    {?statement     ppstatement:hasEffectOn     ?effect .
    ?statement      ppstatement:hasProbiotics   ?probiotics .
    ?statement      ppstatement:hasSpecies      ?species .
    ?statement      ppstatement:hasReference    ?reference .
    ?reference      ppstatement:hasPMID         ?pmid .
    ?probiotics     a                           owl:Class ;
                    owl:intersectionOf          ?mixture .
    ?mixture        rdf:rest*/rdf:first         ?composition .
    ?composition    owl:sameAs                  ?url .
    filter regex    (?effect,"bdnf","i")}
union
    {?url1          rdfs:subClassOf             <http://www.ihtsdo.org/SCT_264395009> .
    ?url1           snomed:hasEnglishLabel      ?urlLabel1 .
    ?sense1         ppconcept:SenseURL          ?url1 .
    ?senses1        ppconcept:hasSense          ?sense1 .
    ?term1          ppconcept:hasSenses         ?senses1 .
    ?annot1         ppconcept:hasLabel          ?termLabel1 .
    ?annot1         ppconcept:hasTerm           ?term1 .
    ?annos1         ppconcept:hasAnnotation     ?annot1 .
    ?annos1         ppconcept:hasSource         ?source1 .
    ?annos1         ppconcept:hasText           ?text1 .
    ?pmid          ppconcept:hasAnnotations    ?annos1 .
    filter regex    (?text1,"bdnf","i")}
    }
```

**SI Listing 4.** TheSPARQL protocol for query case 4: retrieve all probiotics that impact depressive disorder

```
prefix rdf: <http://www.w3.org/1999/02/22-rdf-syntax-ns#>
prefix owl: <http://www.w3.org/2002/07/owl#>
prefix rdfs: <http://www.w3.org/2000/01/rdf-schema#>
prefix snomed: <http://wasp.cs.vu.nl/sct/sct#>
prefix ppconcept: <http://wasp.cs.vu.nl/ppconcept#>
prefix ppstatement: <http://wasp.cs.vu.nl/ppstatement#>

select distinct ?pmid ?probiotics ?regulate ?effect ?composition ?text1 ?text2

where {
    {?statement     ppstatement:hasPopulation   ppstatement:Depressive_disorder ;
                    ppstatement:hasSpecies      ?species ;
                    ppstatement:hasProbiotics   ?probiotics ;
                    ppstatement:hasRegulation   ?regulate ;
                    ppstatement:hasEffectOn     ?effect ;
                    ppstatement:hasReference    ?reference .
    ?reference      ppstatement:hasPMID         ?pmid .}
union
    {?statement     ppstatement:hasPopulation   ppstatement:Depressive_disorder ;
                    ppstatement:hasSpecies      ?species ;
                    ppstatement:hasProbiotics   ?probiotics ;
                    ppstatement:hasRegulation   ?regulate ;
                    ppstatement:hasEffectOn     ?effect ;
                    ppstatement:hasReference    ?reference .
    ?reference      ppstatement:hasPMID         ?pmid .
    ?probiotics     a                           owl:Class ;
                    owl:intersectionOf          ?mixture .
    ?mixture        rdf:rest*/rdf:first         ?composition .}
union
    {?url1          rdfs:subClassOf             <http://www.ihtsdo.org/SCT_264395009> .
    ?url1           snomed:hasEnglishLabel      ?urlLabel1 .
    ?sense1         ppconcept:SenseURL          ?url1 .
    ?senses1        ppconcept:hasSense          ?sense1 .
    ?term1          ppconcept:hasSenses         ?senses1 .
    ?annot1         ppconcept:hasLabel          ?termLabel1 .
    ?annot1         ppconcept:hasTerm           ?term1 .
    ?annos1         ppconcept:hasAnnotation     ?annot1 .
    ?annos1         ppconcept:hasSource         ?source1 .
    ?annos1         ppconcept:hasText           ?text1 .
    ?pmid           ppconcept:hasAnnotations    ?annos1 .
    filter regex    (?text1,"depress","i")}
union
    {?sense2        ppconcept:SenseURL          ?url2 .
    ?senses2        ppconcept:hasSense          ?sense2 .
    ?term2          ppconcept:hasSenses         ?senses2 .
    ?annot2         ppconcept:hasLabel          ?termLabel2 .
    ?annot2         ppconcept:hasTerm           ?term2 .
    ?annos2         ppconcept:hasAnnotation     ?annot2 .
    ?annos2         ppconcept:hasSource         ?source2 .
    ?annos2         ppconcept:hasText           ?text2 .
    ?pmid           ppconcept:hasAnnotations    ?annos2 .
    filter regex(?text2,"depress","i")
    filter regex(?text2,"probiotic","i")}
    }
```
